# Development of a novel analytical pipeline to characterize morphological and molecular features of blood vessels within marginal regions of glioblastoma

**DOI:** 10.1093/noajnl/vdag198

**Published:** 2026-07-30

**Authors:** Anubrata Das, Antoine Vallatos, Joanna Birch, Christopher Ireson, Gerry Thompson, Karen Strathdee, Karin Williams, Christine Littler, Helen Bulbeck, Paul Brennan, Val G Brunton, Colin Smith, Anthony J Chalmers

**Affiliations:** School of Cancer Sciences, University of Glasgow, Glasgow, UK; Glasgow Experimental MRI Centre, School of Psychology and Neuroscience, University of Glasgow, Glasgow, UK; School of Cancer Sciences, University of Glasgow, Glasgow, UK; Pharmidex Pharmaceutical Services Limited, UK; Centre for Clinical Brain Sciences, University of Edinburgh, Edinburgh, UK; School of Cancer Sciences, University of Glasgow, Glasgow, UK; School of Cancer Sciences, University of Glasgow, Glasgow, UK; Brainstrust, Cowes, Isle of Wight, UK; Brainstrust, Cowes, Isle of Wight, UK; Centre for Clinical Brain Sciences, University of Edinburgh, Edinburgh, UK; Edinburgh Cancer Research Centre, University of Edinburgh, Edinburgh, UK; Centre for Clinical Brain Sciences, University of Edinburgh, Edinburgh, UK; School of Cancer Sciences, University of Glasgow, Glasgow, UK

**Keywords:** blood-brain barrier, glioblastoma, multiplex immunohistochemistry, tumor margin

## Abstract

**Background:**

Glioblastomas (GBM) are highly invasive tumors with marginal regions comprising unresectable functional brain infiltrated by tumor cells. Effective drug delivery to these regions is crucial, but lack of understanding of the structural and functional characteristics of their vasculature is impeding drug development. We aimed to develop a bespoke analytical pipeline that could be used to characterize molecular and morphological features of the blood-brain barrier within marginal regions of GBM by analyzing image descriptors extracted from multiplex colorimetric imaging of human samples.

**Methods:**

Multiplex immunohistochemical consecutive staining of key vascular antigens was performed on human samples of GBM and adjacent brain to determine the morphology and composition of blood vessels. A neural network was utilized to segment the vessels, and multiple image descriptors extracted to characterize and classify them with a Linear Discriminant model.

**Results:**

Multiplexed immunohistochemistry was optimized for vessel related-antigens CD31, laminin, claudin-5, smooth muscle actin, platelet-derived growth factor beta, and glial fibrillary acidic protein. Multiple parameters analyzed from the segmented blood vessels were modeled into four distinct categories, linked to region-specific molecular and morphological factors. Margin regions exhibited the lowest vessel density and a heterogenous mix of vessels with some unique to the region and others similar to tumor core or normal brain vessels.

**Conclusions:**

We established a multiplex immunohistochemical staining protocol and pipeline to identify blood vessels and analyze their composition. The pipeline and the preliminary quantitative data it generated will facilitate more comprehensive characterization of margin-specific blood vessels, with implications for drug development.

Key Points

- Multiplex immunohistochemistry reveals morphological and molecular features of GBM vessels.
- Image descriptors distinguish region-specific blood vessels.
- Blood vessels in tumor margins have heterogenous composition.

Importance of the Study

Marginal regions of glioblastomas (GBM) comprise functioning brain infiltrated by sparse tumor cells. Being largely unresectable, these regions are a frequent site of tumor recurrence, hence critical targets for non-surgical treatments. The structure and permeability of blood vessels in marginal regions are poorly defined, making it difficult to design systemic agents that will penetrate them. We used human GBM samples to define a novel analytical pipeline that enabled classification and characterization of vessels in tumor core and margin regions and adjacent normal brain. We observed that margin regions are supplied by region-specific vessels and other “common” vessels that share characteristics with tumor core and normal brain. Margin-specific vessels exhibited irregular distribution of CD31 but uniform staining for laminin, while “common” vessels exhibited irregular morphologies and uneven distribution of CD31, laminin, claudin-5, and platelet-derived growth factor beta. Researchers will be able use the detailed workflow described in the manuscript along with the scripts deposited at GitHub to identify and classify blood vessels in histological images, including multiplex immunohistochemical datasets.

Glioblastoma (GBM) is a lethal primary brain tumor, with patients experiencing early tumor recurrence and death within 12-18 months of treatment comprising surgery, radiotherapy, and temozolomide chemotherapy.^1^ Tumors are highly invasive and generally comprise a solid or partly necrotic tumor core surrounded by a marginal region comprised of relatively normal brain infiltrated by tumor cells at low densities. While neurosurgical debulking of the tumor core provides therapeutic benefits, tumor margin regions are not generally resectable and are therefore the likely source of recurrence and hence mortality for most patients.^2^ It is therefore of critical importance that non-surgical treatments, particularly systemic agents, can be delivered to infiltrative cells in the tumor margins at concentrations sufficient for therapeutic efficacy.

Although the problem of drug delivery to GBM has been recognized for decades, little progress has been made in understanding the factors that determine the extent to which compounds penetrate different regions of these heterogeneous tumors. Sub-optimal drug delivery is frequently attributed to inadequate penetration of the blood-brain barrier (BBB), and drug development for brain tumors has focused largely on maximizing penetration of the normal BBB.^3^ However, this simplistic approach fails to recognize that the BBB is markedly disrupted in GBM, as evidenced by the florid contrast enhancement observed on magnetic resonance (MR) imaging and the histopathological characteristic of microvascular proliferation.^4^^,^^5^ While the molecular and ultrastructural features of the intact BBB have been well described, the characteristics of the “blood-tumor barrier” (BTB) remain poorly understood. Consequently, most preclinical studies utilize simplified models of the intact BBB (eg, MDCK cells expressing MDR1) that fail to reflect the tumor cell infiltration and vascular co-option observed in tumor margin regions of GBM.^6^^,^^7^ The inadequacy of these models is reflected by the lack of efficacy of the therapies they beget. In the phase I OPARATIC trial,^8^ we observed that the poly(ADP-ribose) polymerase (PARP) inhibitor olaparib, which failed to cross the intact BBB in preclinical models, penetrated both core and margin regions in patients with recurrent GBM (median concentrations 496 and 513 nM, respectively) with wide intra- and inter-patient variability and no correlation with blood plasma levels.^8^ The results of this and other “window-of-opportunity” studies indicate that blood vessels in tumor margin regions, although failing to show contrast enhancement on MR imaging, are abnormal and exhibit greater permeability to small molecule therapeutics than vessels in the normal brain.

To date, very little information about the molecular, morphological and functional alterations underlying this increased permeability has been published. Disruption of BBB function in tumor margin regions has been associated with effects of infiltrating tumor cells on astrocyte-vascular coupling^9^ and, more recently, a single cell RNA sequencing study of the GBM vasculature reported differences between tumor and normal brain in terms of gene expression profiles of endothelial cells, pericytes and smooth muscle cells.^10^ To obtain quantitative, clinically relevant data, we undertook multiplex immunohistochemical studies of formalin-fixed, paraffin embedded (FFPE) sections obtained from diagnostic samples of human GBM that included regions of tumor core, margin, and adjacent brain. We then assembled a bespoke analysis pipeline to characterize morphological and molecular features of individual vessels and classify them as belonging to core, margin, or normal brain.

## Methods

### Immunohistochemistry (IHC) of Human GBM and Adjacent Brain Specimens

Resected GBM samples from 3 patients were examined by an experienced neuropathologist who defined morphologically different regions: tumor (core), infiltrating edge of tumor (margin), necrotic tissue (necrotic), and microscopically appearing normal brain (normal). From core, margin and normal brain regions, hematoxylin and eosin (H&E) stained sections were prepared followed by immunohistochemical staining for the key elements of blood-brain (or blood-tumor) barrier: endothelial cells (CD31), inter-endothelial tight junctions (claudin-5), basement membrane (laminin), pericytes (PDGFRβ), astrocytes (GFAP), and smooth muscle cells (SMA). Having performed IHC on individual antigens on serial sections, we then optimized multiplexed immunohistochemistry (mIHC) using the Leica Bond RX fully automated research stainer. This enabled three antibody targets to be identified on a single slide using three different chromogens: DAB (brown), red, and green. The optimization process involved previously characterized antibodies in different concentrations to determine which combinations enabled the most robust analysis. This often required the weakest signal (for example, claudin-5) to be combined with the most prominent chromogen (red). Slides were scanned (Leica Aperio) to create whole scanned images (WSI) that were analyzed.

### Acquisition of Data from IHC Stained Serial Sections of GBM

Stained WSI were accessed in QuPath^11^ and co-registered with the corresponding H&E slide with the Warpy plugin in Fiji.^12^ Then 1024 × 1024 pixels regions of interest (ROI) from regions containing blood vessels were selected for statistical analysis. Custom scripts were written in Fiji, to acquire the DAB channel from the slide and quantify marker expression.

### Acquisition of Data from mIHC Stained Sections and Classification of Vessels

QuPath images were imported into the Warpy plugin of Fiji, and mIHC images co-registered with corresponding H&E sections on which the tumor or margin region has been demarcated by the neuropathologist. We experimented with different image segmentation models from the Segmentation models library^13^ (Supplementary Figure S1B) and selected the UNET framework with efficientnet-b7 autoencoder pretrained on the ImageNet library. The efficientnet-b7 architecture uses compound scaling to store 66 million parameters, which yields a Top-1 accuracy of 84.4%. The UNET framework uses skip connections to re-map the features extracted by the efficientnet-b7 autoencoder to create masks. This UNET framework was trained via transfer learning to identify masks of whole blood vessel cross-sections, stained with three colors, each corresponding to a specific antigen. We then selected 512 × 512 pixel ROIs to scan for blood vessels. Representative sections were used for training, and a dataset of 500 augmented images created using the albumentations library.^14^ Images were resized to 224 pixels for input to the model, which had been pre-trained for this size. The model was trained for 50 epochs until the validation IoU score stabilized at 0.958. The final model was then used to segment masks of blood vessels for further analysis. We optimized a stain matrix to demultiplex the IHC stains using QuPath and used the color deconvolution library of HistomicsTK^15^ to deconvolute the 3-plex stains to individual grayscale images. Label images from the masks were created using the scikit-image library^16^ and algorithms in the HistomicsTK package to extract shape and intensity features and corresponding masks for subsequent analysis. Logistic Regression models and Linear Discriminant Analysis models were applied using the R-package (R version 4.1.2 (2021-11-01). Ordination plots were created using the ggord package.^17^

## Results

### Conventional IHC Characterizes Vessel Morphology but Not Composition in Tumor Margin Regions

**Figure 1.**
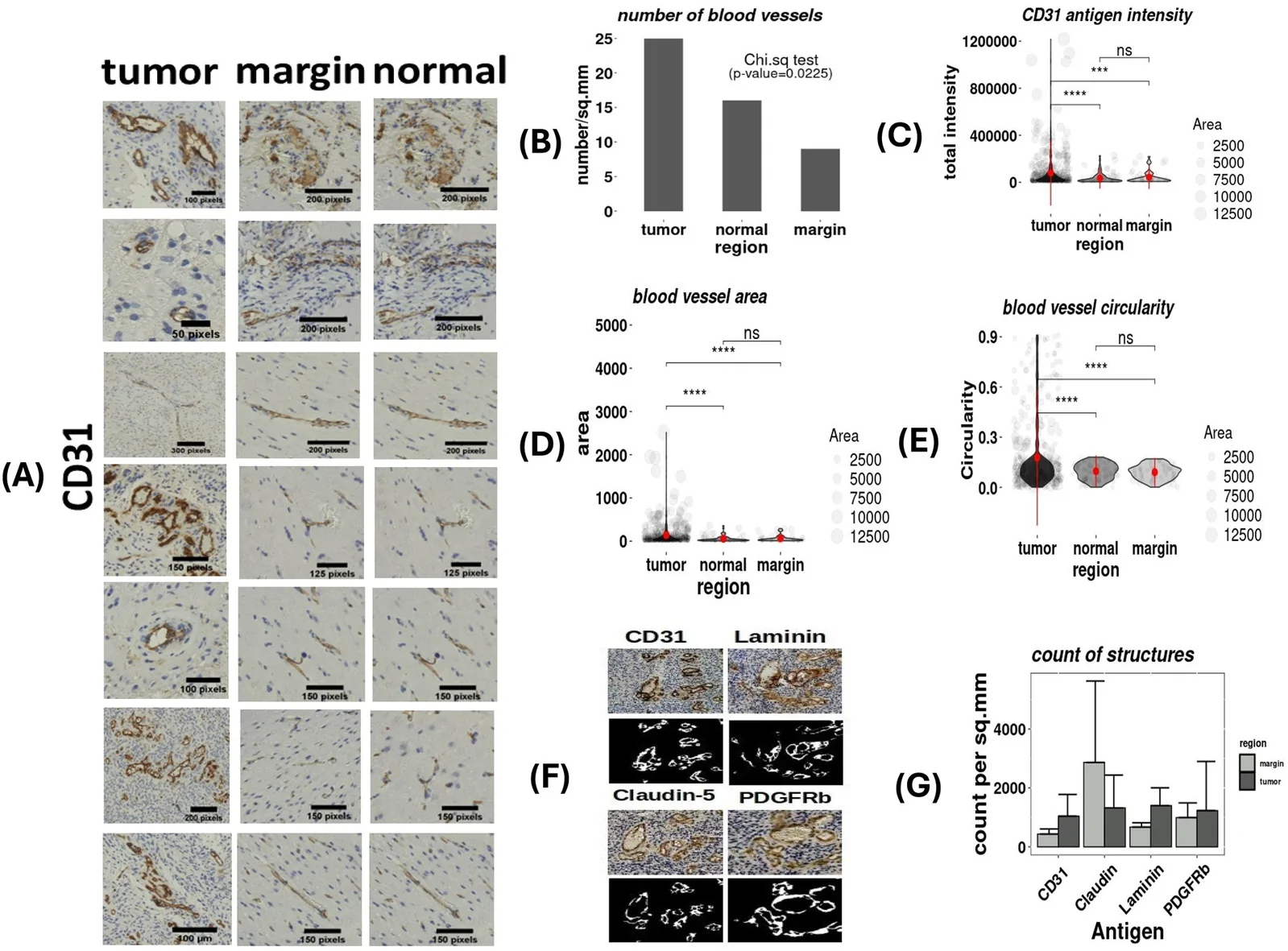
(A) Representative images of human brain sections stained with CD31, claudin-5, PDGFRβ and laminin respectively. (B) Masks were extracted by thresholding CD31 stained structures and the number of structures within selected regions of interest measured. (C) CD31 antigen intensity for each blood vessel plotted for each population. (D) Area occupied by CD31 stain in square pixels in the regions indicated. (E) Circularity of CD31 stained structures in the regions indicated. (F) Representative images and corresponding masks of human brain tissue stained with CD31, claudin-5, PDGFRβ and laminin respectively. Masks were extracted by thresholdin-stained structures and the number of structures within the stained area measured. (G) Stained structures were counted from 1024 × 1024 pixel sized regions of interest (ROI), obtained after co-registering each stained slide with the H&E stained slide annotated by an expert neuropathologist. Structures were counted by Analyze Particles function in ImageJ and plotted. Error bars indicate standard deviation. ****P-value <.0001, ***P-value <.001, ns: not significant. Scale bars are as indicated.

Blood vessels in tumor core, margin and adjacent normal brain were identified by CD31 immunostaining and 1024 × 1024 pixel regions (450.5 × 450.5 µm) were compared for statistical analysis (representative examples shown in Figure 1A). Margin regions exhibited the lowest density of blood vessels (10/mm^2^, Figure 1B(i)) compared with normal brain (15/mm^2^), and tumor core (25/mm^2^). Next, we measured antigen intensity, area, and circularity of the blood vessels and observed that for all parameters tumor core vessels showed greater heterogeneity and range than margin or normal brain vessels. Tumor core vessels expressed higher levels of CD31 (Figure 1C) and were larger, encompassing greater areas (Figure 1D). Tumor core vessels were also more circular, with margin, and normal brain vessels exhibiting narrower cross-sections (Figure 1E). Morphologically, vessels from margin and normal regions were indistinguishable. For further characterization we undertook IHC on consecutive tissue sections from tumor, normal and margin regions, staining for laminin (basement membrane), claudin-5 (tight junctions), platelet derived growth factor beta (PDGFRβ, pericytes) and smooth muscle antigen (SMA, contractility). Despite serial sections being separated by only 4 µm, we found that individual vessels were positionally shifted and exhibited variations in shape between sections. This made it impossible to co-register them without artificially distorting their shape. Hence, we co-registered the outline of the tissue on whole slide images, where the shape was intact across the sections, then selected ROIs from corresponding regions across all serial sections for statistical analysis (Figure 1F). We defined each independent stained object as a structure in these serial sections and observed marked variability between ROIs, as shown in Supplementary Figure S2. While there were trends toward tumor margin ROIs exhibiting lower numbers of structures staining for CD31, laminin, and PDGFRb, but higher numbers of claudin-5 positive structures (Figure 1G), none of these differences were statistically significant. We also noted variability in the area covered by stained structures within each ROI, for both tumor core and margin regions (Supplementary Figure S2). We therefore concluded that while conventional IHC successfully detailed the morphology and number of vessels, it was unable to provide quantitative information on composition of blood vessels in tumor margin regions.

### Multiplexed IHC Reveals Region Specific Characteristics of Blood Vessels

**Figure 2.**
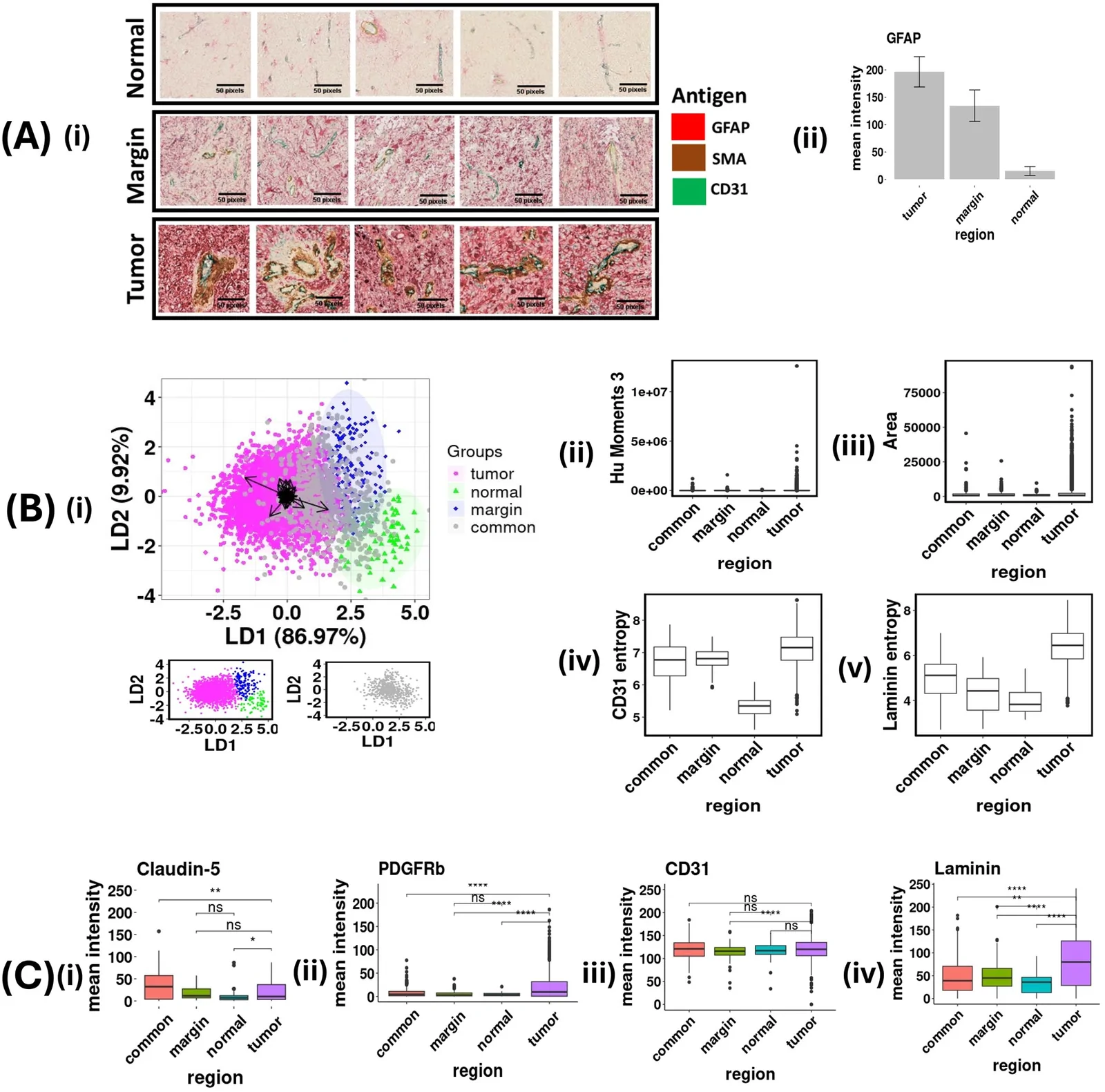
(A) (i) Representative multiplex immunohistochemical images of blood vessels in tumor, margin and normal brain regions. GFAP was labeled with DS9390 Bond Polymer Refine Red Detection, SMA with DAB and CD31 with DC9913 Green Chromogen. (ii) Corresponding pixel intensities of GFAP in the three regions. (B) (i) A Linear Discriminant Analysis (LDA) model separates the blood vessels of the different regions. LD1 and LD2 of the 3 axes are shown. Each point is a blood vessel remapped on the axes after feature extraction. The percentages show the proportion of variance explained by each axis. The plots below separately show separation between blood vessels of the three defined regions and common blood vessels. Boxplot are shown of the region-wise distribution of the following blood vessel features: (ii) Hu Moments 3, (iii) area, (iv) CD31 entropy and (v) laminin entropy. (C) Boxplots depicting the region-wise distribution of the mean intensity of the vasculature antigens (i) claudin-5, (ii) PDGFRβ, (iii) CD31 and (iv) laminin. Error bars indicate standard deviation. ****P-value <.0001, ***P-value <.001, ns: not significant. Scale bars are as indicated.

To enable interrogation of relative antigen expression in each region we carried out multiplexed IHC using three different antibody combinations: (1) CD31, laminin, claudin-5; (b) CD31, laminin, PDGFRβ; and (3) CD31, GFAP, SMA. Representative images are shown in Figure 2A(i). Quantification of the tumor marker GFAP (which is also expressed by astrocytes) validated the neuropathologist’s delineation of tumor, margin, and normal brain regions (Figure 2A(ii)). A bespoke workflow was created to analyze these multiplex IHC images (Supplementary Figure S2). Blood vessels were identified and segmented with a UNET framework based on efficientnetb-7 encoder, which achieved a IoU score of 97% on the test data. The HistomicsTK framework then extracted 46 numerical image descriptors (features) relating to morphology and staining intensities from each of the segmented blood vessels. Since unsupervised classification did not enable discrimination between blood vessels from the different regions, we tested a supervised classifier, Linear Discriminant Analysis (LDA) model,^18^ with the aim of distinguishing blood vessels from normal, margin and tumor core regions. We did not regularize the model but took subsets of the highest ranked features to rebuild it and found that a proportion of vessels was persistently misclassified. When these vessels were removed, accuracy increased from 88% to 96%. A similar approach using Logistic Regression increased accuracy from 83% to 100%, indicating that this phenomenon was model-independent. We therefore assigned these “difficult to classify” vessels to a fourth category, which was labeled “*common*” because it contained vessels that were present in all 3 regions but were distinct from the vessels that were characteristic of each region. An LDA model was then trained to classify vessels into 4 categories: *tumor*, *margin*, *normal,* and *common*. The final model had 90% accuracy (95% CI, 0.9006-0.9143, Figure 2B(i)). The first axis explained ∼87% of the variation and primarily separated tumor vessels from other categories, while the second axis explained ∼10% of the variation and primarily separated normal and margin categories (Figure 2B(i)).

Key features used by the model to discriminate categories were shape features such as Hu Moments (Figure 2B(ii)), area (Figure 2B(iii)), and composition features such as entropy of CD31 distribution (Figure 2B(iv)) and entropy of laminin distribution (Figure 2B(v)). To provide insights into region-specific characteristics of the vessels and to correlate findings from the multiplexed images with the earlier serial sections, we measured the intensities of CD31, laminin, PDGFRβ, and claudin-5. Consistent with the previous findings, vessels in tumor core regions exhibited the highest variation in CD31 intensity followed by the *common* vessels (Figure 2C(i)). Tumor vessels were large (Figure 2B(iii)) with increased levels of irregularly distributed laminin (Figure 2B(v) and C(iv)), in agreement with earlier studies^19^ while vessels in normal brain regions were slender (Figure 2B(iii)) with uniformly distributed CD31 and laminin (Figure 2B(iii) and B(iv)) and low levels of claudin-5 and PDGFRβ (Figure 2C). We also noted increased levels of PDGFRβ in tumor core vessels (Figure 2C(iii)), indicating an increased presence of pericytes.^20^ Based on relative antigen distributions, vessels in tumor margin regions appeared to exhibit irregular distribution of the endothelial layer (high CD31 entropy) but a more uniform basement membrane (low laminin entropy) (Figure 2B(iv and v)) with higher levels of laminin than normal vessels (Figure 2C(iv)). Although previous studies have reported claudin-5 to be transcriptionally downregulated in tumor core vessels, with reduced protein expression in tumour^19^^,^^21^^,^^22^ we observed significant increased claudin-5 intensity in tumor vessels compared with the normal brain vasculature (Figure 2C(i)).

### Blood Vessels With Pleomorphic Identities Predominate in the Margin Region

**Figure 3.**
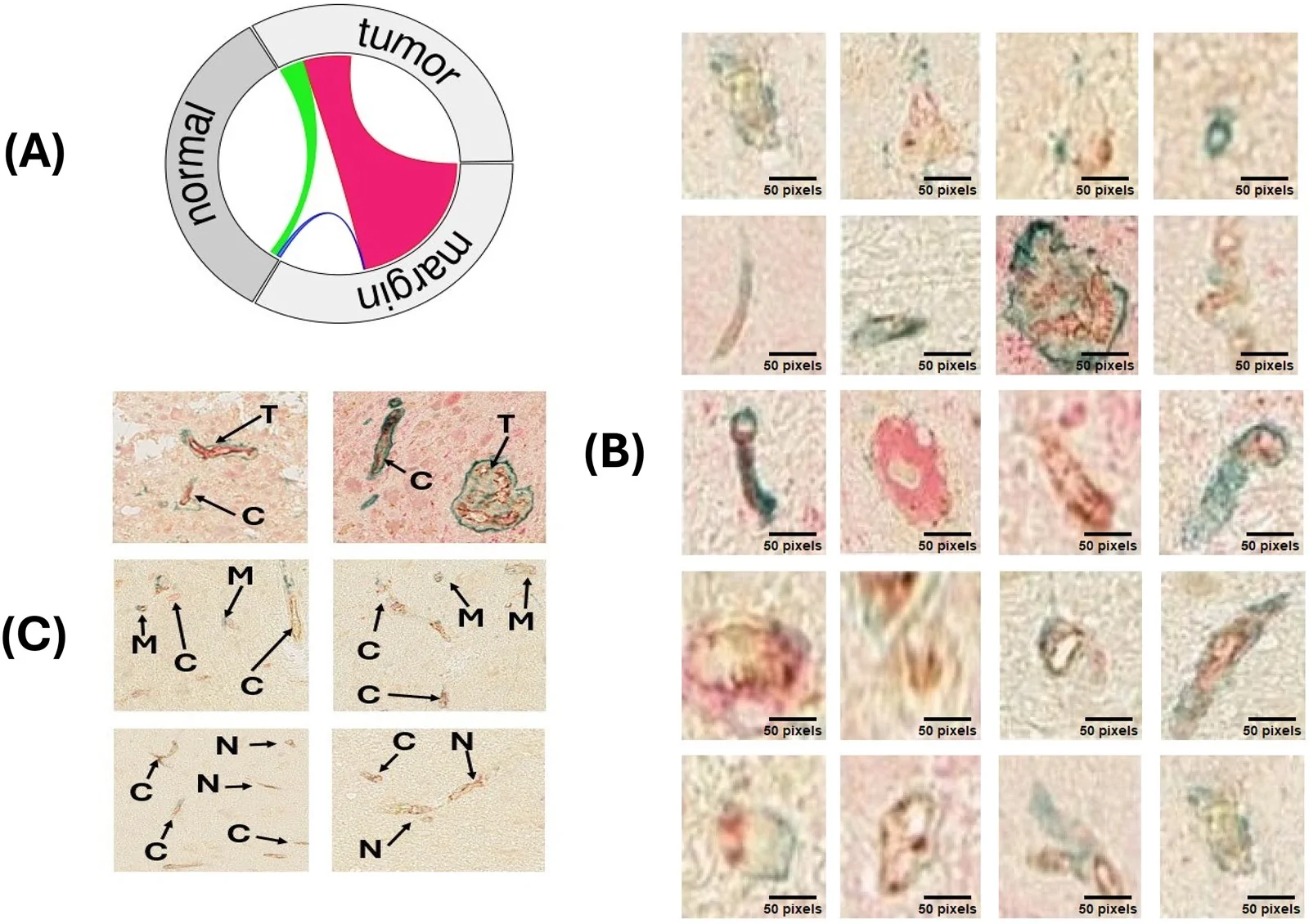
Blood vessels that were persistently difficult to assign to tumor, margin or normal regions by the LDA model were re-classified as “common.” (A) CIRCOS plot showing the proportion of ‘common’ vessels found within the different regions. The red link shows the extent of the similarity between tumor and the margin region vessels, with >50% of vessels located within margin being misclassified as tumor and ∼10% of vessels located in tumor misclassified as margin region vessels. In contrast, <0.5% of margin region blood vessels were mapped to the normal region (blue link) and the ∼1% of vessels in the normal region re-classified as “common” corresponded to 4% of vessels in the tumor region (green link). (B) Representative images of ‘common’ blood vessels. These were located primarily in margin regions but also present in tumor and normal brain. (C) Representative images showing “common” vessels in proximity to (i) tumor region vessels (T), (ii) margin region vessels (M) and (iii) normal region vessels (N). Scale bars are as indicated.

Our model highlighted a category of blood vessels with pleomorphic features that could not be allocated to a specific region (Figure 2B(i)) and were labeled *common* vessels. Although the mean level of claudin-5 expression was highest in these common vessels, substantial inter-vessel variability resulted in high variance (Figure 2C(i)), a property shared with tumors but not margin vessels. We mapped the location of the *common* vessels and observed ∼63% of vessels in margin regions to be of this type, compared with ∼10% of tumor and <1% normal brain vessels (Figure 3A). *Common* vessels exhibited irregular morphologies, with uneven distribution of CD31, laminin, claudin-5, and PDGFRβ (Figure 3B). We analyzed a subset of images to see if the *common* vessels clustered together geographically but found them to be intermixed with margin-specific blood vessels (Figure 3C). Margin regions were thus supplied by a mixture of region-specific vessels and *common* vessels that were also present in tumor, and normal brain, albeit at much lower frequencies. These vessels had irregular distribution of the endothelial antigen CD31 (Figure 2C(i)) and the highest levels of claudin-5 (Figure 2C(i)) when compared to the other classes.

## Discussion

GBM is characterized by extensive abnormalities in the vasculature owing to angiogenesis as well as co-option and physical disruption by infiltrating tumor cells.^23^ Tumor vessels are dilated, torturous, and heterogenous and frequently lack smooth muscle components. As a result, blood flow is reduced and permeability increases. The molecular composition of tumor vessels is also abnormal, with altered expression of vascular endothelial growth factor (VEGF), transforming growth factor beta (TGF-β), matrix metalloproteinases, integrins, and other structural and signaling proteins reported in the literature.^24^ While these morphological and compositional changes increase vascular permeability, poor BBB penetrance remains a major therapeutic problem in GBM and has adversely affected the efficacy of numerous cytotoxic drugs and molecular targeted agents. Considering PARP inhibitors as an example, olaparib, rucaparib, and talazoparib all exhibit poor penetrance of the intact BBB while veliparib has better BBB penetrance with a brain-to-plasma ratio of 0.47 but inferior PARP inhibition and PARP trapping properties that may have contributed to its lack of efficacy in the clinic.^25-29^ To overcome this problem, detailed characterization of the vasculature in both tumor core and tumor margin regions is required, along with pharmacokinetic and pharmacodynamic studies in preclinical models that faithfully represent tumor margin regions as well as tumor core. Immunohistochemical or other proteomic approaches are necessary to define BBB architecture and molecular composition and must be amenable to assessment by neuropathologists as well as detailed statistical analysis.

We approached this challenge by developing a multiplexed IHC protocol that enabled assessment of different regions of tumors and adjacent brain and by creating an analytical pipeline to accurately identify blood vessels, demultiplex the color channels, and extract numerical image descriptors amenable for further statistical analysis. As well as facilitating specific study of tumor margin regions in GBM, we believe that other researchers will be able use the detailed workflow described in the manuscript along with the scripts deposited at GitHub, to identify and classify blood vessels in a range of different histological images including multiplex immunohistochemical datasets.

This approach enabled us to characterize the heterogenous nature of blood vessels in tumor margin regions by integrated analysis of their morphological and molecular features. Despite the caveat that IHC is not quantitative, we were able to distinguish region-specific blood vessels based on quantitative descriptors extracted from the antigenic stains. For example, we observed that normal brain blood vessels exhibit uniform and tightly distributed vascular antigens, as previously reported. Within tumor margin regions we were able to identify vessels unique to those regions as well as vessels sharing similarities with some tumor- or normal-brain vessels that we termed *common* vessels. This heterogeneity likely reflects diffuse and irregular infiltration of marginal regions by GBM cells. Our observation that margin regions shared one type of blood vessel with tumor regions is consistent with previous work that defined 2 types of tumor-related blood vessels: neoangiogenic and co-opted.

Claudin-5 is a key component of the BBB, and reduced claudin-5 expression is associated with increased BBB permeability. We observed that margin regions had blood vessels with 2 distinct patterns of claudin-5 expression: (1) vessels unique to the margin region with lower levels of claudin-5 and (2) *common* vessels with higher claudin-5 levels that were also present in tumor regions. This may be explained by the fact that GBM is associated with local neuroinflammation: exposure of blood vessels to inflammation has been reported to be associated with increased claudin-5 expression.

We also observed that, while tumor vessels exhibited significantly higher levels of laminin than vessels in the normal brain, its distribution showed high levels of entropy, indicating gross irregularity of the basement membrane. High laminin levels have been associated with increased levels of pericytes and vascular smooth muscle cells, and we noted that expression of PDGFRβ, a pericyte marker, was also high in this region.

Based on these findings, we speculate that, in tumor regions, integrity of both the endothelial layer and basement membrane is lost, while in the tumor margin regions, the endothelium is compromised while the basement membrane is preserved. While, in principle, a decision tree classifier could assign every blood vessel to one of the three regions, both our LDA model and an unsupervised principle components analysis (PCA) model were unable to completely segregate between margin and the tumor vessels. This phenomenon is likely explained by the diffusely infiltrative and heterogenous nature of the tumor margin and may also indicate that blood vessels in these regions are in a dynamic state.

While the pipeline described here has enabled us to undertake preliminary characterization of blood vessels in tumor margin regions, it requires validation in samples from an external cohort before routine use. Morphological and molecular data should also be supplemented by detailed, pharmacokinetic, and imaging studies in representative in vivo models to characterize the functional properties of blood vessels in marginal regions of GBM.

## Supplementary Material

vdag198_Supplementary_Data

## Data Availability

The imaging datasets generated in this study will be made available upon reasonable request.

## Code Availability

Computational source codes used for image segmentation, extracting features and LDA analysis have been deposited at GitHub https://github.com/me-nobody/blood_vessel_characterization

## References

[1] Stupp R , Hegi ME, Mason WP, et al; National Cancer Institute of Canada Clinical Trials Group. Effects of radiotherapy with concomitant and adjuvant temozolomide versus radiotherapy alone on survival in glioblastoma in a randomised phase III study: 5-year analysis of the EORTC-NCIC trial. Lancet Oncol. 2009;10:459-466. 10.1016/S1470-2045(09)70025-7 19269895

[2] Ballestín A , Armocida D, Ribecco V, Seano G. Peritumoral brain zone in glioblastoma: biological, clinical and mechanical features. Front Immunol. 2024;15:1347877. 10.3389/fimmu.2024.1347877 38487525 PMC10937439

[3] Karmur BS , Philteos J, Abbasian A, et al Blood-brain barrier disruption in neuro-oncology: strategies, failures, and challenges to overcome. Front Oncol. 2020;10:563840. 10.3389/fonc.2020.563840 33072591 PMC7531249

[4] Karve AS , Desai JM, Gadgil SN, et al A review of approaches to potentiate the activity of temozolomide against glioblastoma to overcome resistance. Int J Mol Sci. 2024;25:3217. 10.3390/ijms25063217 PMC10970334 38542190

[5] Louis DN , Perry A, Reifenberger G, et al The 2016 world health organization classification of tumors of the central nervous system: a summary. Acta Neuropathol. 2016;131:803-820. 10.1007/s00401-016-1545-1 27157931

[6] Jezierza-ski M , Nafalska N, Stopyra M, et al Temozolomide (TMZ) in the treatment of glioblastoma multiforme—a literature review and clinical outcomes. Curr Oncol. 2024;31:3994-4002. 10.3390/curroncol31070296 39057168 PMC11275351

[7] McAleavey PG , Walls GM, Chalmers AJ. Radiotherapy-drug combinations in the treatment of glioblastoma: a brief review. CNS Oncol. 2022;11:CNS86. 10.2217/cns-2021-0015 35603818 PMC9134931

[8] Hanna C , Kurian KM, Williams K, et al Pharmacokinetics, safety, and tolerability of olaparib and temozolomide for recurrent glioblastoma: results of the phase I OPARATIC trial. Neuro Oncol. 2020;22:1840-1850. 10.1093/neuonc/noaa104 32347934 PMC7746945

[9] Watkins S , Robel S, Kimbrough IF, Robert SM, Ellis-Davies G, Sontheimer H. Disruption of astrocyte-vascular coupling and the blood-brain barrier by invading glioma cells. Nat Commun. 2014;5:4196. 10.1038/ncomms5196 24943270 PMC4127490

[10] Xie Y , Yang F, He L, et al Single-cell dissection of the human blood-brain barrier and glioma blood-tumor barrier. Neuron Published. 2024;112:3089-3105. 10.1016/j.neuron.2024.07.026 39191260

[11] Bankhead P , Loughrey MB, Fernández JA, et al QuPath: open source software for digital pathology image analysis. Sci Rep. 2017;7:16878. 10.1038/s41598-017-17204-5 29203879 PMC5715110

[12] Schindelin J , Arganda-Carreras I, Frise E, et al Fiji: an open-source platform for biological-image analysis. Nat Methods. 2012;9:676-682. 10.1038/nmeth.2019 22743772 PMC3855844

[13] Iakubovskii P. Segmentation Models. GitHub repository. 2019. https://github.com/qubvel/segmentation_models. Date accessed May 17, 2025.

[14] Buslaev A , Iglovikov VI, Khvedchenya E, Parinov A, Druzhinin M, Kalinin AA. Albumentations: fast and flexible image augmentations. Information (Switzerland). 2020;11:125. 10.3390/info11020125

[15] Amgad M , Beezeley J, Chittajallu DR, et al HistomicsTK. GitHub Repository. 2016. https://github.com/DigitalSlideArchive/HistomicsTK. Date accessed May 17, 2025.

[16] Van Der Walt S , Schönberger JL, Nunez-Iglesias J, et al; scikit-image contributors. Scikit-image: image processing in Python. PeerJ. 2014;2:e453. 10.7717/peerj.453 25024921 PMC4081273

[17] Beck MW. ggord: ordination plots with ggplot2. Preprint posted online 2024.

[18] Zhao S , Zhang B, Yang J, Zhou J, Xu Y. Linear discriminant analysis. Nat Rev Methods Primers. 2024;4:70. 10.1038/s43586-024-00346-y

[19] Liebner S , Fischmann A, Rascher G, et al Claudin-1 and claudin-5 expression and tight junction morphology are altered in blood vessels of human glioblastoma multiforme. Acta Neuropathol. 2000;100:323-331. 10.1007/s004010000180 10965803

[20] Cheng L , Huang Z, Zhou W, et al Glioblastoma stem cells generate vascular pericytes to support vessel function and tumor growth. Cell. 2013;153:139-152. 10.1016/j.cell.2013.02.021 23540695 PMC3638263

[21] Karnati HK , Panigrahi M, Shaik NA, et al Down regulated expression of claudin-1 and claudin-5 and up regulation of b-catenin: association with human glioma progression HHS public access. Vol 13. 2014. 10.2174/1871527313666141023121550 PMC6138250 25345514

[22] Ling Y , Kang X, Yi Y, Feng S, Ma G, Qu H. CLDN5: from structure and regulation to roles in tumors and other diseases beyond CNS disorders. Pharmacol Res. 2024;200:107075. 10.1016/j.phrs.2024.107075 38228255

[23] Rosi-ska S , Gavard J. Tumor vessels fuel the fire in glioblastoma. Int J Mol Sci. MDPI. 2021;22. 10.3390/ijms22126514 PMC8235363 34204510

[24] Fukumura D , Jain RK. Tumor microvasculature and microenvironment: targets for anti-angiogenesis and normalization. Microvasc Res. 2007;74:72-84. 10.1016/j.mvr.2007.05.003 17560615 PMC2100036

[25] Kizilbash SH , Gupta SK, Chang K, et al Restricted delivery of talazoparib across the blood–brain barrier limits the sensitizing effects of PARP inhibition on temozolomide therapy in glioblastoma. Mol Cancer Ther. 2017;16:2735-2746. 10.1158/1535-7163.MCT-17-0365 28947502 PMC5716902

[26] Obrador E , Moreno-Murciano P, Oriol-Caballo M, et al Glioblastoma therapy: past, present and future. Int J Mol Sci. 2024;25:2529. 10.3390/ijms25052529 PMC10931797 38473776

[27] Parrish KE , Cen L, Murray J, et al Efficacy of PARP inhibitor rucaparib in orthotopic glioblastoma xenografts is limited by ineffective drug penetration into the central nervous system. Mol Cancer Ther. 2015;14:2735-2743. 10.1158/1535-7163.MCT-15-0553 26438157 PMC4674360

[28] Xiong Y , Guo Y, Liu Y, et al Pamiparib is a potent and selective PARP inhibitor with unique potential for the treatment of brain tumor. Neoplasia. 2020;22:431-440. 10.1016/j.neo.2020.06.009 32652442 PMC7350150

[29] Yuan AL , Ricks CB, Bohm AK, et al ABT-888 restores sensitivity in temozolomide resistant glioma cells and xenografts. PLoS One. 2018;13:e0202860. 10.1371/journal.pone.0202860 30153289 PMC6112648

