# Supplementary material for: Development of a novel analytical pipeline to characterize morphological and molecular features of blood vessels within marginal regions of glioblastoma
Source: Neurooncol Adv. 2026 Jul 30;8(1):vdag198. doi: 10.1093/noajnl/vdag198 (PMC13525839; doi:10.1093/noajnl/vdag198)
Supplement: Supplementary file 1 [file vdag198_supplementary_data.zip › Das -GM-BBB Supplementary Figure Legends.docx]

**Das *et al*. Supplementary Figure Legends**

**Supp Fig S1.** Workflow for analysis of multiplex images. (A) slides were stained with (1) DS9390 Bond Polymer Refine Red Detection (2)  DAB- 3,3'-diaminobenzidine (3) DC9913 Green Chromogen for vasculature antigens. Images of the slides were processed as indicated. NN model – UNET framework based pre-trained neural network model. HistomicsTK- Python based image analysis framework. (B) Development of a deep learning model to identify blood vessels from multiplex IHC data. A pre-trained segmentation models library was trained with pathologist identified blood vessel data. Initially several encoders, as indicated, were experimented for the UNET framework (C) The pre-trained model was optimized for highest accuracy and (D ) learning rate and dropout rate optimized.

**Supp Fig S2.** Stained area occupied by IHC antigens in the serial sections. As mentioned, in results, representative 1024 x1024 pixel ROI were chosen from co-registered WSI images, and the area occupied by each stained structure in the ROI was measured. Non-parametric Kruskal Wallis test was done to estimate the within margin and within tumor variation between ROI and non-parametric Wilocoxon rank sum test was done to measure variation between margin and tumor regions, as shown in the figure  (A) DAB stained serial section of CD31 (B) DAB stained serial section of laminin (C ) DAB stained serial section of PDGFRb (D) DAB stained serial section of Claudin-5
